# Can Pretreatment Serum Beta-hCG be Used for Predicting Thyrotoxicosis in Gestational Trophoblastic Disease?

**DOI:** 10.31557/APJCP.2021.22.11.3461

**Published:** 2021-11

**Authors:** Uraiwan Khomphaiboonkij, Chanisorn Termsarasab

**Affiliations:** *Department of Obstetrics and Gynecology, Lampang Hospital, Lampang, Thailand. *

**Keywords:** Gestational trophoblastic disease, thyrotoxicosis, beta-hCG

## Abstract

**Background:**

Gestational trophoblastic disease (GTD) comprises a diverse spectrum of entities of abnormal cellular proliferations originating in placental trophoblasts. The specific marker of GTD is beta-hCG which has a similar structure to the TSH molecule, interfering level of thyroid hormones. How and when to check for thyroid function test during this period remain challenging.

**Objective:**

To assess values of pretreatment beta-hCG and its benefit for predicting thyrotoxicosis among patients with diagnoses of GTD.

**Methods:**

Retrospective analytical study included all women diagnosed with GTD at Lampang Hospital from January 2010 to May 2020. The patients’ pretreatment beta-hCG and thyroid function were collected. Sensitivity and specificity for detecting laboratory hyperthyroidism were reported and classified by pretreatment beta-hCG levels.

**Results:**

Forty-four women with diagnoses of GTD were recruited. The range of pretreatment beta-hCG levels were classified into 4 groups: beta-hCG > 50,000 IU/ml (group 1), beta-hCG > 100,000 IU/ml (group 2), beta-hCG > 150,000 IU/ml (group 3), beta-hCG > 200,000 IU/ml (group 4). The sensitivity for prediction of high fT4 were 100%, 94.1%, 94.1% and 88.2% in group 1,2,3 and 4, respectively, while the specificity were 12%, 20%, 32% and 44% in group 1,2,3 and 4, respectively.

**Conclusion:**

Pretreatment beta-hCG > 100,000 uIU/ml has the high sensitivity and acceptable specificity for predicting hyperthyroidism. So we don’t need to check or wait for thyroid function test in patients who had beta-hCG < 100,000 IU/ml.

## Introduction

Gestational trophoblastic disease (GTD) comprises a diverse spectrum of entities of abnormal cellular proliferations originating in placental trophoblasts. GTD is classified into two categories, hydatidiform mole or molar pregnancy (complete and partial) and gestational trophoblastic neoplasia (GTN). The term GTN has been applied to three pathologic malignant conditions: invasive mole, choriocarcinoma, and intermediate trophoblastic tumors such as placental site trophoblastic tumor (PSTT) or epithelioid trophoblastic tumor (ETT).

The incidence of molar pregnancy has been reported at 0.6 – 1.1 per 1,000 pregnancies in North America, Australia, New Zealand and Europe but it is two to three-fold higher in South East Asia and Japan (2.0 per 1,000 pregnancies) (Schink and Lurain, 2017).

The most accurate disease specific marker of GTD is hCG, which is produced by hydatidiform moles (Schink and Lurain, 2017). The hCG molecule is made of α and β subunits, which have similar structures to the TSH molecule. Since hCG and TSH receptors are similar, hCG acts directly on the TSH receptors that are present in the thyroid resulting in an increased level of thyroid hormones and decreased TSH levels.

The large case series comes from Africa (Norman et al.,1981). In 27 patients with GTD, more than half (15 out of 27) were biochemically hyperthyroid at presentation. The study from UK reviewed of the 196 patients with gestational trophoblastic neoplasia, 14 (7%) had biochemical hyperthyroidism (Walkington et al., 2011).

Galton (1971) reported the first thyrotoxicosis case in a woman with a hydatidiform mole. The spectrum of thyroid function changes varies from slight increases in freeT4 (fT4) and free T3 (fT3) and low TSH levels with no thyrotoxicosis symptoms, to moderate increases in fT4 and fT3 and up to increases large enough to cause clinical thyrotoxicosis (Kenimer et al.,1975) . In approximately 67% of the cases, beta-hCG levels greater than 200,000 mIU/ml have been found to suppress TSH (lower or equal to 0.2 mIU/ml), while levels above 400,000 mIU/ml can lead to suppression in up to 100% of cases and it is estimated that for every 10,000 mIU/ml increase in serum beta-hCG, TSH decreases by 0.1 mlU/ml and free T4 increases by 0.1 ng/dL (Glinoer, 1997). Depending on the severity of the trophoblastic disease, the patient may have clinically asymptomatic elevations of thyroid hormones or can have something as severe as thyrotoxicosis. 

Suction evacuation and curettage is the preferred method of evacuation of a molar pregnancy independent of uterine size if maintenance of fertility is desired. Hysterectomy is an alternative to suction curettage if childbearing is complete (Bhatla and Denny, 2018). In patients with uncontrolled hyperthyroidism, surgery and anesthesia are associated with significant perioperative mortality (Virmani et al., 2017). Hence it is important to evaluate for thyroid function in every patient of GTD in order to anticipate and be prepared for complications that may occur during a surgical procedure. 

As Kofinas (2015) reported thyroid storm in a patient of GTD, which had the values of fT4 of 4.9 ng/ml, TSH of 0.06 uIU/ml and beta hCG of 1,488,021 IU/ml while a case report in 2019 found the value of fT4 of 1.82 ng/ml, TSH of 0.009 uIU/ml and beta hCG of 117,495 IU/ml (Blick and Schreyer, 2019). Meanwhile, Wie (2016) reported a thyroid storm in a partial hydatidiform mole woman, who had beta-hCG 1,046,900 mIU/mL, fT4 3.06 ng/ml and T3 1.96 pg/ml. The degree of increased thyroid hormone varies. The potency of beta-hCG for TSH receptors is some 4000 times less than TSH and hence, extremely high levels of beta-hCG are usually required for an effect on thyroid function to be seen (Norman et al.,1981). 

Given the prevalence and potential dangers of thyroid storm, many have suggested universally evaluating thyroid function in all gestational trophoblastic disease women before treatment. However, the cost, effectiveness, and practical nature of any such approach must be taken into account. How and when to check for thyroid function test, how and if to treat thyroid illness during this period remains challenging.

As is often the case with rare tumors, research and advances are rarely publicized. So the objective of this study was to assess the values of pretreatment beta-hCG and its benefit for predicting thyrotoxicosis among patients with diagnoses of gestational trophoblastic disease at our hospital over the past 10 years. 

## Materials and Methods

The institutional review board of Lampang hospital approved the study protocol (registration number 045/2020). Informed consent was waived due to retrospective nature of data collection. All collected data were de-identified and kept confidential in compliance with Declaration of Helsinki. 

Our retrospective analytical study included all women diagnosed with gestational trophoblastic disease who had undergone treatment and had received further evaluation and management at Lampang Hospital from January 2010 to May 2020. The diagnoses were confirmed by their medical record and their biochemical markers which were reviewed from our computerized system.

*Data collection*

The patient’s age, number of pregnancies, interval of antecedent pregnancy, histology and treatment methods were recorded. The patient’s pretreatment beta-hCG, TSH, fT4 and fT3 were collected. Hyperthyroid was diagnosed with a thyroid level elevated more than the normal reference value (fT3: 1.710 – 3.710 pg/ml, fT4: 0.77 – 1.48 ng/dl) or TSH level was lower than the normal reference value (TSH: 0.350 – 4.940 uIU/ml).

*Statistical analysis*

Statistical analysis of the data was carried out using Stata version 14.1. Demographic data was determined using percentage, mean, standard deviation and interquartile range. Sensitivity and specificity for detecting laboratory hyperthyroidism were reported and classified by pretreatment beta-hCG values.

*Objective*

To assess values of pretreatment beta-hCG and its benefit for predicting thyrotoxicosis among patients with diagnoses of gestational trophoblastic disease at our hospital over the past 10 years. 

## Results

During the study period, 44 women with diagnoses of gestational trophoblastic disease were recruited. The demographic data of the patients were reported in Table 1.

The mean age was thirty one. Eleven patients were first-time pregnancy . The history of term pregnancy was the most common found (47.7%). The median time of antecedent pregnancy was 54 months . 

According to the pathology results from 44 gestational trophoblastic diseased patients included in this study, 33 patients (75%) were diagnosed with a complete mole. There was one patient (2.3%) each with histology of a partial mole and choriocarcinoma, meanwhile 9 patients (20.5%) had no data.

Regarding the laboratory results of hyperthyroidism (low TSH, High fT4 and high fT3) among the patients in this study, twenty-eight patients (63.6%) were diagnosed hyperthyroid by low TSH level. Eighteen patients (40.9%) and twenty-two patients (50%) had high level of fT4 and fT3, respectively.

The patients were grouped according to the pathology results as presenting a benign disease (complete and partial molar pregnancy) or a malignant disease (choriocarcinoma). Initial laboratory thyroid function values were reported in Table 2. Nine patients were missed due to unknown histology. One patient was diagnosed as choriocarcinoma who had a low level of fT4 (0.56 ng/ml), normal fT3 (3.39 pg/ml) and normal TSH level (1.58 uIU/ml). Thirty-four patients were diagnosed as molar pregnancy. Their median fT4 value was 1.41 ng/ml, fT3 value was 3.845 pg/ml and TSH value was 0.1 uIU/ml.

The range of pretreatment beta-hCG levels were 18,721 to 7,600,000 uIU/ml in molar pregnancy with a median value of 491,242 uIU/ml. Pretreatment beta-hCG level was not recorded in the choriocarcinoma patient and the other one who had unknown histology. There were 3 patients with beta-hCG levels below 50,000 uIU/ml , 3 patients with beta-hCG levels 50,000 – 100,000 uIU/ml, 3 patients with beta-hCG levels 100,000 – 150,000 uIU/ml, 4 patients with beta-hCG levels 150,000 – 200,000 uIU/ml and 29 patients with beta-hCG levels above 200,000 uIU/ml.

In order to determine the pretreatment beta-hCG level, which can be used as predictor for hyperthyroidism, forty-two patients who had the recording of the pretreatment beta-hCG and thyroid function value were classified into 4 groups: beta-hCG > 50,000 uIU/ml (group 1), beta-hCG > 100,000 uIU/ml (group 2), beta-hCG > 150,000 uIU/ml (group 3), beta-hCG > 200,000 uIU/ml (group 4). We evaluated the sensitivity and specificity of each group to assess the usefulness for hyperthyroid diagnoses in Table 3. 

There were 17 out of 42 patients which had a high levels of fT4. The sensitivity for prediction of high fT4 levels were 100%, 94.1%, 94.1% and 88.2% in group 1,2,3 and 4, respectively.

Twenty one out of 42 patients had high levels of fT3. The sensitivity for prediction of high fT3 levels were 95.2%, 90.5%, 85.7% and 85.7% in group 1,2,3 and 4, respectively.

There were 27 out of 42 patients which had low TSH. The sensitivity for prediction of low TSH levels were 100%, 96.3%, 96.3% and 88.9% in group 1, 2, 3 and 4, respectively.

**Table 1:** Demographic Data

	Missing data	N=44	
	n (%)	n	(%)
Age (year, mean±SD)	3 (6.8)	31.4	±11.7
Antecedent pregnancy	7 (15.9)		
Term		21	-47.7
Molar		0	0
Abortion		5	-11.4
Nulliparous		11	-25
Interval of antecedent pregnancy (month, median (IQR)	20 (45.5)	54	-24,156
Histology			
Complete mole	9 (20.5)	33	75
Partial mole		1	2.3
Choriocarcinoma		1	2.3
PSTT		0	0
ETT		0	0
Laboratory hyperthyroid			
Abnormal fT4	0 (0)	18	40.9
Abnormal fT3	0 (0)	22	50
Abnormal TSH	0 (0)	28	63.6

SD, standard deviation; IQR, interquartile range; PSTT, placental site trophoblastic tumor; ETT, epithelioid trophoblastic tumor; fT4, free T4; fT3, free T3; TSH, thyroid-stimulating hormone.

**Table 2:** Initial Laboratory Values for Patients

	n	median	IQR
FT4 (ng/dl)			
Molar	34	1.41	0.97, 2.15
GTN	1	0.56	0.56, 0.56
FT3 (pg/ml)			
Molar	34	3.845	2.95, 4.82
GTN	1	3.39	3.39, 3.39
TSH (uIU/ml)			
Molar	34	0.1	0.03, 0.4
GTN	1	1.58	1.58, 1.58

IQR, interquartile range; fT4, free T4; fT3, free T3; TSH, thyroid-stimulating hormone; GTN, gestational trophoblastic neoplasia.

**Table 3:** Thyroid Function Test Classify by Beta-hCG

Beta-hCG	Abnormal	Normal	Sensitivity (95%CI)	Specificity (95%CI)
FT4				
≥50,000	17	22	100	12
<50,000	0	3	(80.5-100)	(2.55-31.2)
≥100,000	16	20	94.1	20
<100,000	1	5	(71.3-99.9)	(6.83-40.7)
≥150,000	16	17	94.1	32
<150,000	1	8	(71.3-99.9)	(14.9-53.5)
≥200,000	15	14	88.2	44
<200,000	2	11	(63.6- 98.5)	(24.4- 65.1)
Total	17	25		
FT3				
≥50,000	20	19	95.2	9.52
<50,000	1	2	(76.2-99.9)	(1.17-30.4)
≥100,000	19	17	90.5	19
<100,000	2	4	(69.6-98.8)	(5.45-41.9)
≥150,000	18	15	85.7	28.6
<150,000	3	6	(63.7–97)	(11.3-52.2)
≥200,000	18	11	85.7	47.6
<200,000	3	10	(63.7–97)	(25.7-70.2)
Total	21	21		
TSH				
≥50,000	27	12	100	20
<50,000	0	3	(87.2–100)	(4.33-48.1)
≥100,000	26	10	96.3	33.3
<100,000	1	5	(81-99.9)	(11.8-61.6)
≥150,000	26	7	96.3	53.3
<150,000	1	8	(81-99.9)	(26.6-78.7)
≥200,000	24	5	88.9	66.7
<200,000	3	10	(70.8-97.6)	(38.4-88.2)
Total	27	15		

beta-hCG, beta-subunit human chorionic gonadotropin hormone; fT4, free T4; fT3, free T3; TSH, thyroid-stimulating hormone

## Discussion

Our result show that GTD affects women in the mean age 31 years. In previous study (Rachdi et al., 2019), the median age was 34 years, which is in comparison with our study. In our study, the TSH range in gestational trophoblastic disease patients was 0.03 – 0.4 uIU/ml., which is the same range published in the American Thyroid Association guidelines 2017 describing a downward shift of the TSH reference range occurring during normal pregnancy, with a reduction in both the lower (decreased by about 0.1–0.2 mU/L) and the upper limit of maternal TSH (decreased by about 0.5–1.0 mU/L), relative to the typical nonpregnant TSH reference range (Alexander et al., 2017). The TSH reference range in the first trimester was 0.02-3.78 mU/L on Nanjing study (Zhang D, 2019). It suggests that the TSH range in our gestational trophoblastic diseased patients had the same range of normal pregnant woman. One case diagnosed as choriocarcinoma had a little higher level of TSH (1.58 uIU/ml ) but the beta-hCG level was not recorded in this patient, This is the limitation of our study.

Free T4 and free T3 reference ranges in our patients were 0.77 – 1.48 ng/dl and 1.710 – 3.710 pg/ml, respectively. While the normal pregnant range of fT4 is 0.75 – 1.39 ng/dl (Bestwick et al., 2014). Our result shows fT4 range 0.97 – 2.15 ng/dl and FT3 range 2.95 - 4.82 pg/ml. It seems that fT3 maybe interfered by illness more than FT4.

One out of 3 patients had a beta-hCG level below 50,000 IU/ml was found with laboratory hyperthyroidism by fT3 value 4.47 pg/ml. Two out of 3 patients had beta-hCG levels between 50,000 -100,000 uIU/ml, the first one had fT4 value 1.5 ng/dl and the other had fT3 value 4.82 pg/ml. They did not receive the thyroid storm prevention and had no complications during or after treatment.

In the previous study Lockwood et al., (2009), during pregnancy TSH was suppressed in 67% of the specimens with hCG concentrations >200,000 IU/L while our study shows 82.8% (24/29), which could reflect the differences within the population. Among the population of the previous study, 19% had GTD. As Norman (1981) described that the serum beta-hCG > 100,000 mIU/ml are usually needed to produce the clinical evidence of thyrotoxicosis. 

In our cohort study, pretreatment beta-hCG > 100,000 uIU/ml demonstrated a sensitivity and specificity for predicting elevated fT4 were 94.1% and 20 %, respectively. Among these patients there was 90.5% sensitivity and 19% specificity for predicting elevated fT3, which demonstrated a high sensitivity and an acceptable specificity. There has not been any report before referencing sensitivity and specificity of beta-hCG for predicting thyroid dysfunction, so these results could have significant benefits in practical use. 

Removal of the hydatidiform mole and appropriate chemotherapy for the choriocarcinoma lead to a rapid resolution of hyperthyroidism (Galton et al., 1971; Hershman et al.,1971) and should be done as soon as possible. But a thyroid storm is a life-threatening condition that should raise concern and awareness of the certainty. In the literature reviews, most thyroid storm patients had a beta-hCG levels more than 200,000 mIU/ml (Erbil et al., 2006; Hwang et al., 2014; Kofinas et al., 2015; Wie et al.,2016; Swaminathan et al., 2017; Moskovitz et al., 2020).

The lowest beta-hCG level was 117,495 mIU/ml which occurred in a thyroid storm case from Philadelphia (Blick and Schreyer, 2019). From our study, gestational trophoblastic diseased patient who had a beta-hCG value less than 100,000 mIU/ml rarely found laboratory confirmed hyperthyroidism. 

Nowadays early pregnancy sonograms and easy availability of beta-hCG titers have led to more timely diagnoses of gestational trophoblastic disease, with an ensuing dramatic reduction in the incidences of associated hyperthyroidism (biochemical or clinical)(Misra et al.,2002). In our opinion , if the patient has pretreatment beta-hCG levels less than 100,000 mIU/ml, we don’t need to check or wait for any thyroid function investigations, so we can safely do the suctional and curettage in this population.

Limitation of this study is that each patient was not carefully examined for signs of hyperthyroidism and incomplete medical records.

In conclusion, in gestational trophoblastic diseased patients, pretreatment beta-hCG levels > 100,000 uIU/ml has the high sensitivity and acceptable specificity for predicting laboratory hyperthyroidism. Most patients with pretreatment beta-hCG levels < 100,000 uIU/ml lack overt laboratory hyperthyroidism so we don’t need to check or wait for thyroid function test in these patients.

*Highlights*

• Gestational trophoblastic disease may induce thyrotoxicosis that requires treatment.

• Beta-hCG levels can predict laboratory hyperthyroidism.

• Treatment of GTD can initiate regardless of thyroid function test, if beta-hCG levels less than 100,000 mIU/ml.

## Author Contribution Statement

Uraiwan Khomphaiboonkij, Chanisorn Termsarasab performed acquisition of patient data, analysis and interpretation of patient data. Uraiwan Khomphaiboonkij developed the study conception and design. Uraiwan Khomphaiboonkij performed critical revision of the manuscript. All authors have read and agreed to the final version of this manuscript.

## Ethics approval and consent to participate

Approval was obtained from The institutional review board of Lampang hospital to participate to the study.

## Competing interests

The authors declare no competing interest.

## References

[B1] Alexander EK, Pearce EN, Brent GA (2017). 2017 Guidelines of the American Thyroid Association for the diagnosis and management of thyroid disease during pregnancy and the postpartum. Thyroid.

[B2] Bestwick JP, John R, Maina A (2014). Thyroid stimulating hormone and free thyroxine in pregnancy: expressing concentrations as multiples of the median (MoMs). Clin Chim Acta.

[B3] Bhatla N, Denny L (2018). FIGO Cancer Report 2018. Int J Gynaecol Obstet.

[B4] Blick C, Schreyer KE (2019). Gestational trophoblastic disease-induced thyroid storm. Clin Pract Cases Emerg Med.

[B5] Erbil Y, Tihan D, Azezli A (2006). Severe hyperthyroidism requiring therapeutic plasmapheresis in a patient with hydatidiform mole. Gynecol Endocrinol.

[B6] Galton VA, Ingbar SH, Jimenez-Fonseca J, Hershman JM (1971). Alterations in thyroid hormone economy in patients with hydatidiform mole. J Clin Invest.

[B7] Glinoer D (1997). The regulation of thyroid function in pregnancy: pathways of endocrine adaptation from physiology to pathology. Endocr Rev.

[B8] Hershman JM, Higgins HP (1971). Hydatidiform mole--a cause of clinical hyperthyroidism Report of two cases with evidence that the molar tissue secreted a thyroid stimulator. N Engl J Med.

[B9] Hwang W, Im D, Kim E (2014). Persistent perioperative tachycardia and hypertension diagnosed as thyroid storm induced by a hydatidiform mole: a case report. Korean J Anesthesiol.

[B10] Kenimer JG, Hershman JM, Higgins HP (1975). The thyrotropin in hydatidiform moles is human chorionic gonadotropin. J Clin Endocrinol Metab.

[B11] Kofinas JD, Kruczek A, Sample J, Eglinton GS (2015). Thyroid storm-induced multi-organ failure in the setting of gestational trophoblastic disease. J Emerg Med.

[B12] Lockwood CM, Grenache DG, Gronowski AM (2009). Serum human chorionic gonadotropin concentrations greater than 400,000 IU/L are invariably associated with suppressed serum thyrotropin concentrations. Thyroid.

[B13] Misra M, Levitsky LL, Lee MM (2002). Transient hyperthyroidism in an adolescent with hydatidiform mole. J Pediatr.

[B14] Moskovitz JB, Bond MC (2010). Molar pregnancy-induced thyroid storm. J Emerg Med.

[B15] Norman RJ, Green-Thompson RW, Jialal I (1981). Hyperthyroidism in gestational trophoblastic neoplasia. Clin Endocrinol (Oxf).

[B16] Rachdi H, Mokrani A, Batti R (2019). Gestational Trophoblastic Neoplasia: A Tunisian Multicenter Study. Asian Pac J Cancer Care.

[B18] Swaminathan S, James RA, Chandran R, Joshi R (2017). Anaesthetic implications of severe hyperthyroidism secondary to molar pregnancy: a case report and review of literature. Anesth Essays Res.

[B19] Virmani S, Srinivas SB, Bhat R, Rao R, Kudva R (2017). Transient thyrotoxicosis in molar pregnancy. J Clin Diagn Res.

[B20] Walkington L, Webster J, Hancock BW, Everard J, Coleman RE (2011). Hyperthyroidism and human chorionic gonadotrophin production in gestational trophoblastic disease. Br J Cancer.

[B21] Wie JH, Kwon JY, Ko HS (2016). Thyroid storm and early-onset proteinuric hypertension caused by a partial molar pregnancy. J Obstet Gynaecol.

[B22] Zhang D, Cai K, Wang G (2019). Trimester-specific reference ranges for thyroid hormones in pregnant women. Medicine (Baltimore).

